# The BinDiscover database: a biology-focused meta-analysis tool for 156,000 GC–TOF MS metabolome samples

**DOI:** 10.1186/s13321-023-00734-8

**Published:** 2023-07-20

**Authors:** Parker Ladd Bremer, Gert Wohlgemuth, Oliver Fiehn

**Affiliations:** 1 Department of Chemistry, University of California, Davis, CA 95616 USA; 2 West Coast Metabolomics Center for Compound Identification, UC Davis Genome Center, University of California, Davis, CA 95616 USA

**Keywords:** Metabolomics, Gas chromatography, Mass spectrometry, Meta-analysis, Ontologies

## Abstract

Metabolomics by gas chromatography/mass spectrometry (GC/MS) provides a standardized and reliable platform for understanding small molecule biology. Since 2005, the West Coast Metabolomics Center at the University of California at Davis has collated GC/MS metabolomics data from over 156,000 samples and 2000 studies into the standardized BinBase database. We believe that the observations from these samples will provide meaningful insight to biologists and that our data treatment and webtool will provide insight to others who seek to standardize disparate metabolomics studies. We here developed an easy-to-use query interface, BinDiscover, to enable intuitive, rapid hypothesis generation for biologists based on these metabolomic samples. BinDiscover creates observation summaries and graphics across a broad range of species, organs, diseases, and compounds. Throughout the components of BinDiscover, we emphasize the use of ontologies to aggregate large groups of samples based on the proximity of their metadata within these ontologies. This adjacency allows for the simultaneous exploration of entire categories such as “rodents”, “digestive tract”, or “amino acids”. The ontologies are particularly relevant for BinDiscover’s *ontologically grouped differential analysis*, which, like other components of BinDiscover, creates clear graphs and summary statistics across compounds and biological metadata. We exemplify BinDiscover’s extensive applicability in three showcases across biological domains.

**Supplementary Information:**

The online version contains supplementary material available at 10.1186/s13321-023-00734-8.

## Introduction

Metabolomics databases can serve a variety of purposes. Some databases compile spectral libraries into repositories that users can download and incorporate into their identification workflows. Examples include MassBank of North America (https://massbank.us) [1] or Global Natural Products Social Molecular Networking (GNPS) [2]. Other examples include study-centric databases that store the metadata and observations of user-submitted studies, including the Metabolomics Workbench [3], MetaboLights, and ReDU [3–5] databases. Others, such as the Human Metabolite Database (HMDB) [6], can be loosely described as information compilers, as they synthesize information from a range of sources. Finally, but not exhaustively, are compilation databases, that aggregate multiple, smaller, databases. A recent example of this is the COCONUT (COlleCtion of Open Natural ProdUcTs) database for natural products [7]. One of the most tantalizing research directions in metabolomics is harmonizing the archipelago of datasets in order to create a critical mass of synergistic data that can be used to achieve broad understanding of biology [8]. Within the MetabolomicsWorkbench database, users can query metabolite-centric comparisons with Venn diagrams and metabolite ratios data tables. While such queries are easily performed for individual compounds, navigating interfaces for bulk queries across different study designs is best performed via application programming interfaces (APIs) that require computational expertise. HMDB compiles information from disparate sources. HMDB and related databases from the same laboratory are reliable because the information is manually curated in painstaking efforts. For both HMDB and MetabolomicsWorkbench queries, meta-analysis on bulk metabolite queries suffers because it is a retrospective attempt to harmonize compound-centric information sets across multiple biological study designs. Too much biological metadata is lost in translating either text sources (as in HMDB) or cryptic and unstructured sample/treatment naming schemes (as used during MetabolomicsWorkbench uploads). At least for compound names, MetabolomicsWorkbench employs a database-internal naming scheme, RefMet. However, neither confidence levels for compound annotations nor concentration values are known for MetabolomicsWorkbench, due to the complexity and variety of instrument conditions.

Within individual laboratories, data may be more harmonized due to use of a specific type of instrumentation under defined protocols. Here, tools like meta-XCMS [9] or Amanida [10] allow for the generation of results that come from multiple studies. However, such tools expect a specific input data format, and such data files are not homogeneous even within a laboratory when different individuals process metabolomics raw data. Hence, even on a laboratory level, gathering data in a systematic way to render compiled results accessible to meta-analyses tools is not straightforward. Hence, classic meta-analysis is performed on a higher abstract level such as pathways or reducing to sets of synonymous names [11], instead of queries of bulk metabolite tables.

We recognize the challenge of aggregating results derived across labs and methods. We therefore posit that standardization of protocols is key to useful cross-study comparisons and queries, for both study metadata and data acquisition processes. Here, standard operating procedures are more mature in GC–MS metabolomics compared to LC–MS/MS. At UC Davis, we operate a unified, automated workflow to process metabolomics data since 2005, called BinBase. We here took a snapshot of all data processed until winter 2021 to enable large scale, multi-study meta analyses to investigate the data. We term this tool BinDiscover. It is a webtool to enable users to perform meta-analysis within minutes to extract data trends and propose hypotheses. Rather than simply comparing two types of metadata (e.g. two different species with the same organ), we assigned all metadata into ontologies to empower broad comparisons such as phylo organizations or *ontologically grouped differential analysis* (OGDA). OGDA queries transform broad questions into sets of smaller categories and then combine statistical result outputs into graphs.

## Methods

The BinDiscover database draws spectral and compound information from the GC-Binbase database [12–14]. GC-Binbase uses a bucket sort approach, where new peaks from the chromatographic runs of samples are either matched to previously annotated groupings or identified as new compounds. This bucket sort is algorithmic, with a retention index tolerance of 2000 Fiehn RI units based on fatty acid methyl ester internal standards (FAME), accounting for approximately 2 s absolute retention time windows, and matching unique ions that are determined during the MS deconvolution process. Weighted dot product similarity scores are used to match new experimental data against Bins in GC-BinBase using different signal purity and signal intensity thresholds [14]. All compound annotations have been manually conducted and curated over the past 20 years. Additional details such as automatic recognition of ‘isomeric interferences’, ‘peak purity’, ‘peak apex ions’, ‘unique ion’, ‘signal/noise’ and further parameters indicating data quality are used within GC-BinBase as output by the vendor’s ChromaTOF software that was used for MS-deconvolution [12–14]. Spectra presented in BinDiscover are consensus spectra that constantly improve spectra quality for all individual mass spectra that are assigned to a Bin (a mass spectrum with a specific unique ion and a specific retention index).

For generating the BinDiscover database, all analyses were conducted using custom python scripts that are available in Github (see “Data availability”). We heavily employed statistics routines from SciPy and the network analysis framework from NetworkX. Development was performed locally before full-data transformation on a 64 core, 128-GB RAM Amazon Web Services (AWS) virtual machine. The BinDiscover output database is deposited on a Postgres database managed by AWS. The API employed the Flask library and the frontend relied heavily on Plotly/Dash. The API and frontend were containerized with docker and deployed on AWS Elastic Beanstalk.

## Results

### BinBase is an automatic data processing database for GC–TOF mass spectrometry

**Fig. 1:**
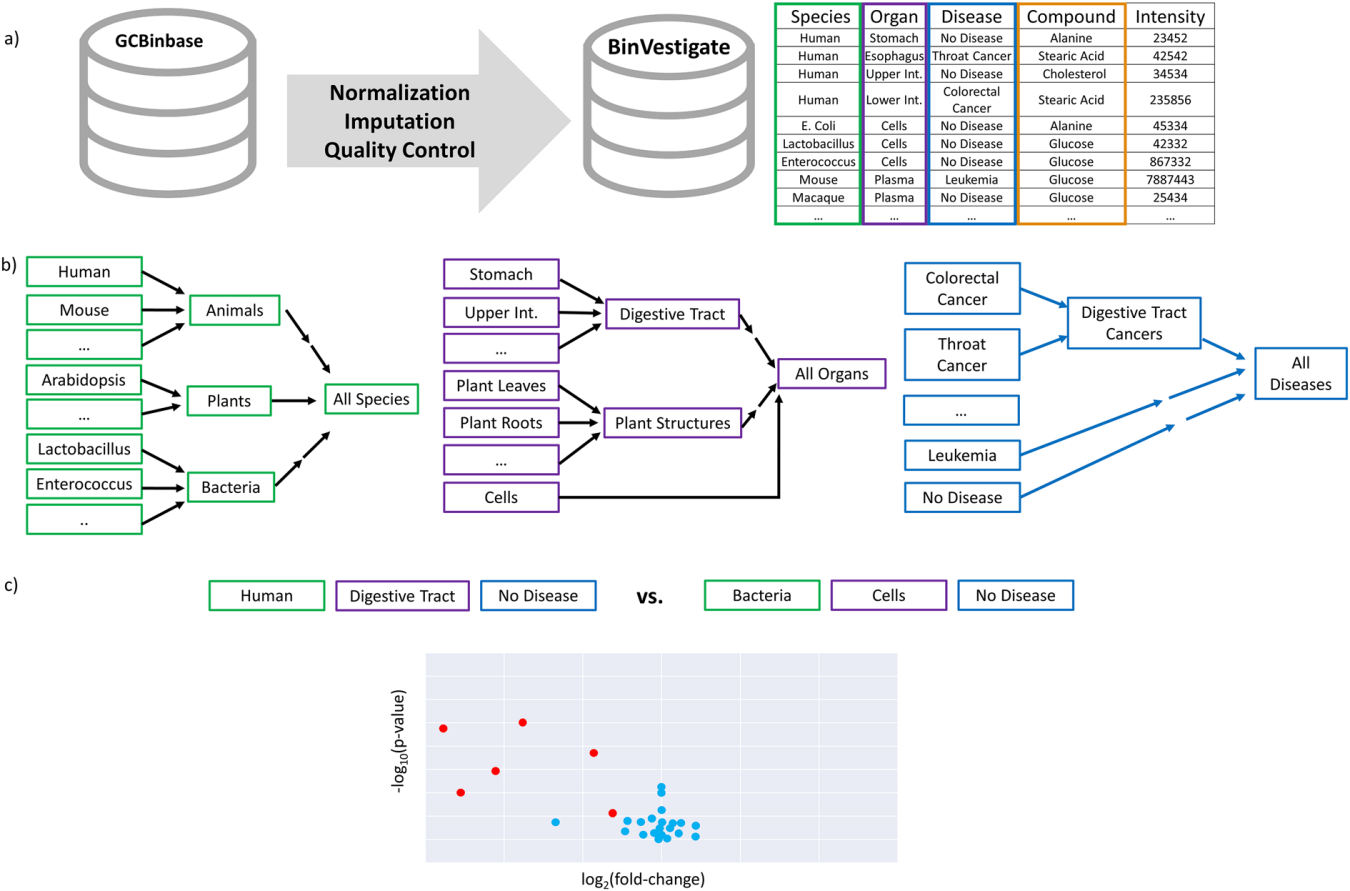
Overall workflow for BinDiscover database queries. **a** BinBase records observations from 156,174 metabolomic samples run on a GC–TOF mass spectrometer from 2005 to 2021. Corresponding biological metadata were curated and the resulting annotation table formed the basis of the exploratory webtool BinDiscover. **b** BinDiscover associates metabolite intensities across species, organs, and diseases. Established ontologies are used to order biological metadata for queries. For metabolites, we used the ClassyFire ontology to enable compound class-level queries. **c** Biological metadata are associated with all samples and are represented and can be queried via different ontology levels, such as “digestive system” or “bacteria”. Species, organ and disease ontologies are highlighted by colors

At the UC Davis West Coast Metabolomics Center, primary metabolites are studied for 18 years using identical workflows for data acquisition and data processing using gas chromatography-time of flight mass spectrometry (GC–TOF MS). At current, five GC–TOF MS instruments are in operation. Standard operating procedures have been published extensively and have been locked and remained unchanged since 2005. Data were aligned by a set of fatty acid methyl ester internal standards, forming a stable retention index. Co-eluting mass spectra were deconvoluted and automatically de-noised by the instruments’ software. This software also provided a range of metadata on the quality of data reports, from peak purity to isomeric interferences, absolute and relative ion intensities, and unique ions that best described the presence of specific metabolites within the proximity of other compounds. All this metadata was utilized by a multi-level filtering algorithm to generate a comprehensive database for both known and unidentified metabolites, called BinBase. To query biological metadata for cross-study analyses, we downloaded all data from BinBase in December 2021. This data comprised 156,174 samples that were processed into 18,290 Bins, i.e. unique mass spectra at specific retention times that used specified quantification ions. Bins included 773 identified metabolites, 39 known chemical artifacts (like polysiloxanes that originate during the GC–TOF MS process) and 15,843 spectra that were not annotated as specific chemicals. The remaining bins were accounted for by, over the course of 17 years of use, algorithmic artifacts that led to multiple bins which were merged into single metabolite values during data exports. Some Bins are associated with the same biological metabolite due to incomplete chemical trimethylsilylation, as has been reported before. We generated a workflow to investigate the biological associations for each Bin, called BinDiscover. A simplified workflow is shown in Fig. 1. GC–TOF MS Compound identifications were performed within the BinBase administrative graphical user interface (GUI) (BinView) using both mass spectral spectral similarity and retention index difference between library spectra and calculated retention times. For compound identification, the FiehnLib library [12] was used in conjunction with MassBank.us and NIST20 spectra [15]. Kovats retention index values (based on alkane elution order) were automatically normalized to Fiehn retention indices that are based on fatty acid methyl ester (FAME) elution order.

### Wrangling and transforming metabolomic and biological metadata

Each Bin is associated with biological information with respect to all studies when it was positively detected. Biological metadata were curated as detailed below, mapping sample metadata to established ontologies. We used three ontologies: (1) the National Center for Biotechnology Information (NCBI) taxonomy for species [16, 17], (2) the Medical Subject Headings (MeSH) taxonomy for organs and diseases [18], and the ClassyFire ontology for compounds [19]. In total, we used and input of 1696 metadata combinations, defined as specific organ/species/disease triad. Across all samples, a total of 55,261,308 observed metabolites were associated with Bins, along with the full spectra and intensities of the quantification ions for each specific Bin. Each sample in BinBase is associated with information on the corresponding biological study that was conducted. Studies included both published and unpublished experiments, as data were gathered for both in-house academic purposes over the past 18 years, as well as for extramural fee-for-service projects. Biological metadata was entered into the small version of SetupX, called miniX [13]. Clients entered minimal information such as species, organs, short abstracts and sample labels that contained text for specific aspect of study designs. Since there is no universal algorithm to capture all details of biological designs in coherent and machine readable forms, the biological metadata necessarily remained heterogeneous. We therefore had to curate biological metadata and transform and normalize ion intensities.

**Fig. 2:**
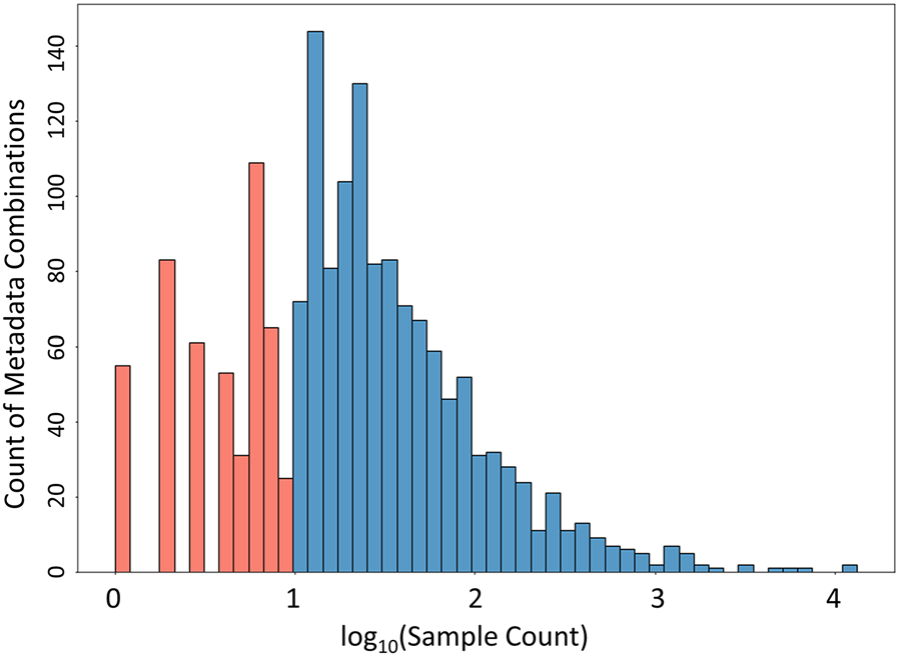
Sample count for all combinations of biological metadata triads. Triads with fewer than 10 samples (red) were removed to increase statistical reliability

The first step was to remove technical variance that arose from using four GC–TOF mass spectrometers and varying instrument conditions over the last 17 years. Across all studies, the exact same concentrations of FAME internal standards were used, offering us the opportunity to use FAME retention index markers as a surrogate value for instrument performance for each specific sample. Hence, we normalized metabolite intensities in each sample by the sum of the FAME ion intensities. We validated that FAME intensities showed correlations greater than 0.8 across all samples, demonstrating that they also reflected differences in GC–MS injection conditions. Next, we automatically identified problematic samples and excluded those from BinDiscover. To do this, we removed samples with poor FAME patterns, as defined as extremely high or low FAME intensity values. In addition, we removed entire biological metadata triads if they showed more than 20% failed FAME samples (Additional file 3: Fig. S1), or if there were fewer than 10 samples in total for a specific biological metadata triad class. This data wrangling ensured that outliers did not have outsized effects on average metabolite intensities for any specific biological class. In this way, we balanced maximizing metadata coverage and maintaining statistical reliability. The distribution of sample counts is shown in Fig. 2. Next, we curated and combined metadata combinations to map metadata to established ontologies and to correct for misspellings. Metadata were manually entered into miniX over the last 17 years, leading to an array of metadata combinations for ‘homo sapiens’, ’homo sapien’, ‘Human’, ‘human’, spellings with extra spaces or tabs, and different synonyms for either species or organs. All strings were transformed into formal ontology entries, accounting for the largest reduction of metadata combinations. Overall, 515 metadata combinations remained, concomitant with a 23.3% reduction of specimen to a total number of 119,783 samples. The next type of data wrangling accounted for correcting intensity values for unique bins. Here, we first combined bins that were best represented by a single unique metabolite. Such double bins arose over the course of 17 years because of multiple derivatization forms (with or without trimethylsilylation of amino groups) or because of incorrect retention time index calculations due to overloaded chromatograms. To obtain a single intensity for each compound for each sample, we preferentially drew intensities from the most-populated bin. If that bin was not detected, we scaled the intensity of the next-most-populated bin according to the average intensity ratio between the two bins. Overall, we retained 16,616 bins to be associated with the metadata combinations (773 metabolites with known chemical structure, and 15,843 unknowns).

Lastly, we had to impute missing values. Here, we considered four scenarios (Additional file 3: Fig. S2). (1) A specific bin might be truly absent from a sample, and perhaps even from a full metadata combination. Indeed, most bins were absent from most biological specimens, for biological reasons. However, when calculating intensity ratios of bins between organs or species, ratio fold-changes become infinite when compounds are absent from one organ or species but present in the other. (2) On the other hand, a bin might be absent is a sample due to random errors, such as thresholds in peak detection algorithms. For example, as reported before, our BinBase algorithm uses conservative thresholds for spectral quality based on signal intensity. If a peak failed weighted dot-score similarity thresholds of 700, that bin would not be declared to be found in that sample, and missed in the BinBase database. Manual investigations or recursive backfilling might find such peak, but those approaches are not tractable. We call such peaks missing at random (MAR), while truly missing compounds (for biological reasons) can be thought of as missing not at random (MNAR). (3) Most peaks are not found 100% of all samples in a specific metadata combination, or 0% detected, i.e. always absent, but somewhere in-between. Imputing the minimum intensities for missing data has been shown to work well for MAR metabolomics data using vectors of samples or vectors of features [20]. However, if a bin is largely absent for a specific metadata combination (i.e. very rarely detected), a single outlier could grossly inflate the overall distribution. Therefore, we imputed the percentage of presence, multiplied by the minimum value of detected peaks (bins) for each metadata combination. In this way, if nearly all samples have annotations, then we simply impute the minimum. If nearly all samples lack annotations, then we impute a small number that is close to the noise level and will conserve the semi-quantitative fold change. This approach also provides a solution to the uncommon, but challenging case of ~ 50% present, where the data neither clearly represent MAR nor MNAR cases. (4) Lastly, if a bin is completely absent, there is no minimum value. In this case, we imputed a value such that the average for any 0% MDC will appear on the left edge of the average distribution for that compound across all metadata combinations (such that differential analysis would show an increase from the 0% case). Hence, for all bins and all metadata combination, a value is given, often as a small noise term. After normalizing, imputing, and curating distributions for all bins and all metadata combinations, we calculated derivatives of the bin intensities to empower comparisons and queries of metabolome-wide metadata combinations. Here, we calculated the averages, medians, and ratios of intensity values and stored the resulting dataset in a PostgreSQL BinDiscover database. We also computed the Welch t-test on pairs of log-transformed pairs of distributions. We chose log-transformed data here instead of directly using Welch t tests due to the known phenomenon of typically non-Gaussian distributions of metabolite values. The results of fold-change and significance calculations were stored, rather than the underlying distributions, in order to dramatically speed-up the return of query results in real-time for user queries.

**Fig. 3:**
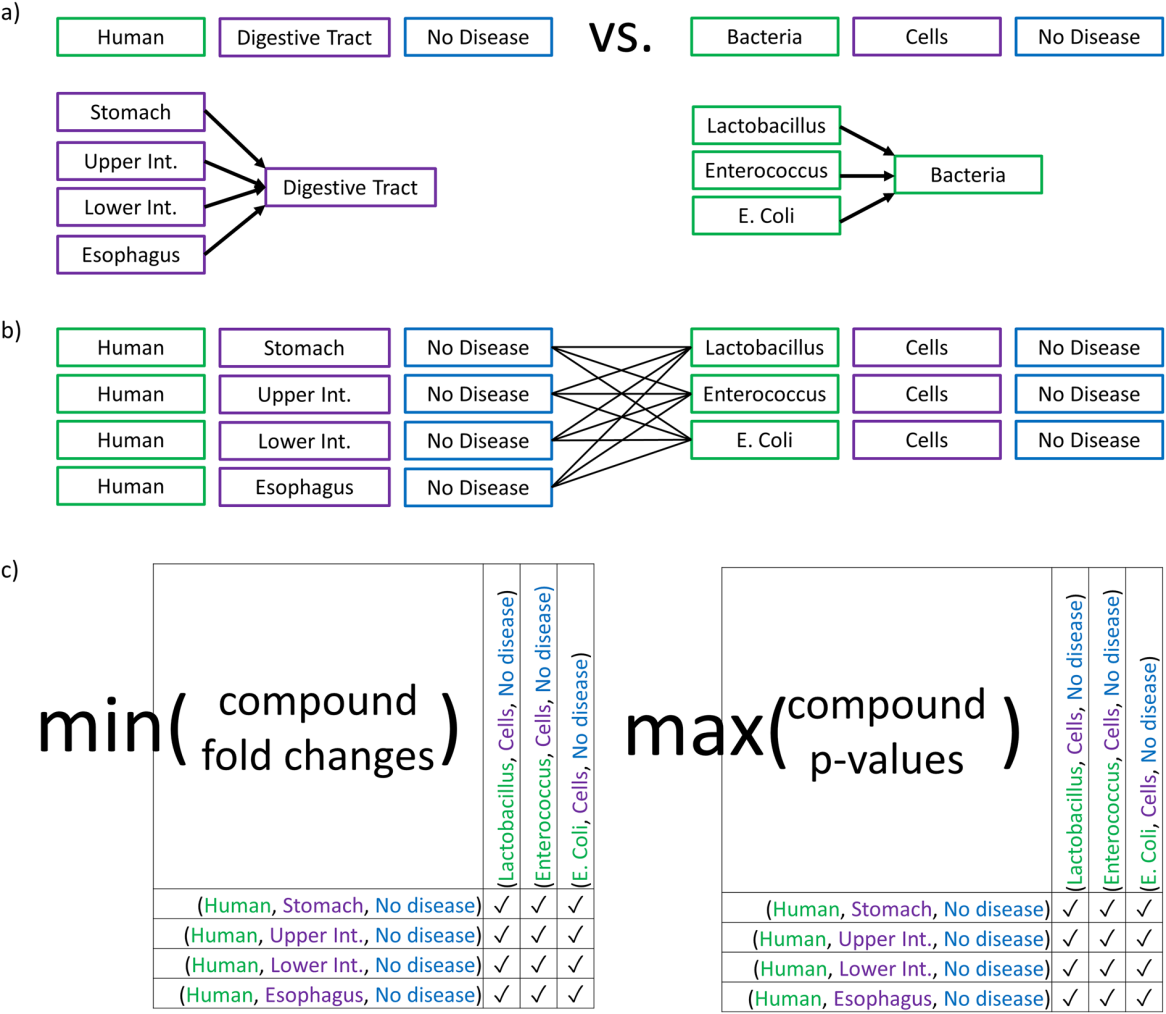
Schema for ontologically grouped differential analysis. Example query human digestive tract versus bacterial metabolomes. **a** All BinBase samples with metadata that ontologically map to (Human, Digestive System without Disease) were compared to samples that mapped to (Bacteria Cells without Disease). **b** Such ontology-based summary queries yield a set of biological metadata combinations that are then subjected to pairwise differential analysis. **c** For each compound, pairwise differential analysis yields a matrix of p-values and a matrix of fold changes that can be conservatively described by the maximum p-value and minimum fold-change, respectively. Therefore, only one point is visualized per compound in downstream volcano plots

### Ontologically grouped differential analysis

We here introduce *ontologically grouped differential analysis* (OGDA) to extract generalizations hidden within the complex data in Omics databases. In metabolomics as well as proteomics or genomics databases, studies performed by biologists or biomedical scientists comprise complex study designs that ultimately can be described in biological metadata that are associated with each sample. We summarized the biological metadata that was available to us using Medical Subject Header (MeSH) ontologies, ClassyFire chemical ontologies and NCBI species ontologies. Hence, all sample metadata were tabularized into ontological sets. OGDA then exploits ontologies to select sets based on their taxonomic proximities. In this way, samples from many studies can be compared on different ontological hierarchies on a database-wide level. Hence, intractable lists of results get transformed into condensed lists to base further analysis.

To exemplify the power of this approach, we randomly used three use cases involving queries on organ levels across species, queries across species, queries on a human disease level, and queries on metabolite levels. Figure 3 demonstrates how ontologically grouped differential analyses calculations are performed. Here, a nutritional researcher might be interested in querying the metabolomic differences between microbial cells (bacteria) and metabolites that are found in the human digestive tract. Hence, the example query would use the BinDiscover ontology triads [(Human, Digestive Tract, no Disease) vs. (Bacteria, Cells, no Disease)] on a very generic term level (Digestive Tract and Bacteria) that by themselves would not be found in the study metadata. Yet, such words and abstractions are commonly used and understood in the literature.

To process this request, BinDiscover transforms the given request into an equivalent request that utilizes all relevant and available samples within BinBase. The ontology search yields all samples that associated with ‘Digestive Tract’ or ‘Bacteria’ and obtains a set of all nodes that are ontologically related to the requested hierarchical level (“belongs to”). Details are given in Additional file 3: Table S1. Importantly, stool (human feces) does not belong to the MeSH ontology of digestive system, but to the ontology “fluids and secretions”. Hence, human stool samples were not included in this specific query. We then summarize all samples and transform the higher ontology level request into a list of related metadata combinations. The metabolomes of all BinBase samples that are summarized to the query groups defined in this manner are then subjected to pairwise statistical analyses. For each pair, BinDiscover creates classic results of a list of Welch-test statistical *p*-values and corresponding fold-changes between the two query sets. Therefore, if we have *n* combinations for one ontology sample set and *m* combinations for the comparator sample set, we yield n*m fold-changes and p values for each metabolite. The results can then be rethought of as an n*m fold change matrix and an n*m p-value matrix for every compound (Fig. 3c).

Next, BinDiscover simplifies these compound matrices to exactly one aggregated *p*-value and associated fold-change for each compound. To extract overarching trends across the database, we conservatively estimate results for each compound across all n*m pairs. For example, if at least one bacterium showed significant higher levels of a metabolite than any human gut organ, but other bacteria would not be significant, this metabolite would not be summarized as an overall significant difference between bacterial metabolism and human gut samples. To maintain this level of conservative constraint, we therefore used the maximum *p*-value for each compound and the minimum fold change as boundaries. If statistical tests were overall significant, but n*m pairs showed both positive and negative fold-changes, BinDiscover represents the fold change as 0. For the example query shown in Fig. 3, we ultimately did not find any chemically identified bacterial metabolite that was significantly different and at higher levels than detected in human gut metabolomes. However, the query retrieved 15 significant metabolites that were found in increased levels in human digestive system organs (Additional file 3: Table S2). These compounds can be summarized into vitamins, lipids, sterols, and amino acid derivatives. These metabolites are indeed not known to be directly produced by bacteria but relate to human food metabolism in a broad sense, confirming the validity of BinDiscover queries to match classic information that could be derived from scientific literature. When we conducted tests for the 773 structurally identified compounds, we obtained results in 26 s, at a rate of approximately 1 s per metadata combination query. When we repeated the analyses for 15,843 unknown compounds, BinDiscover retrieved results in 8 min and 40 s, at a rate of 6.9 s per query. Overall, we found 74 unknown compounds to be at significantly higher levels in bacteria, and 0 compounds in higher concentrations in human organs.

### Case study 1—exploring food metabolomes

**Fig. 4:**
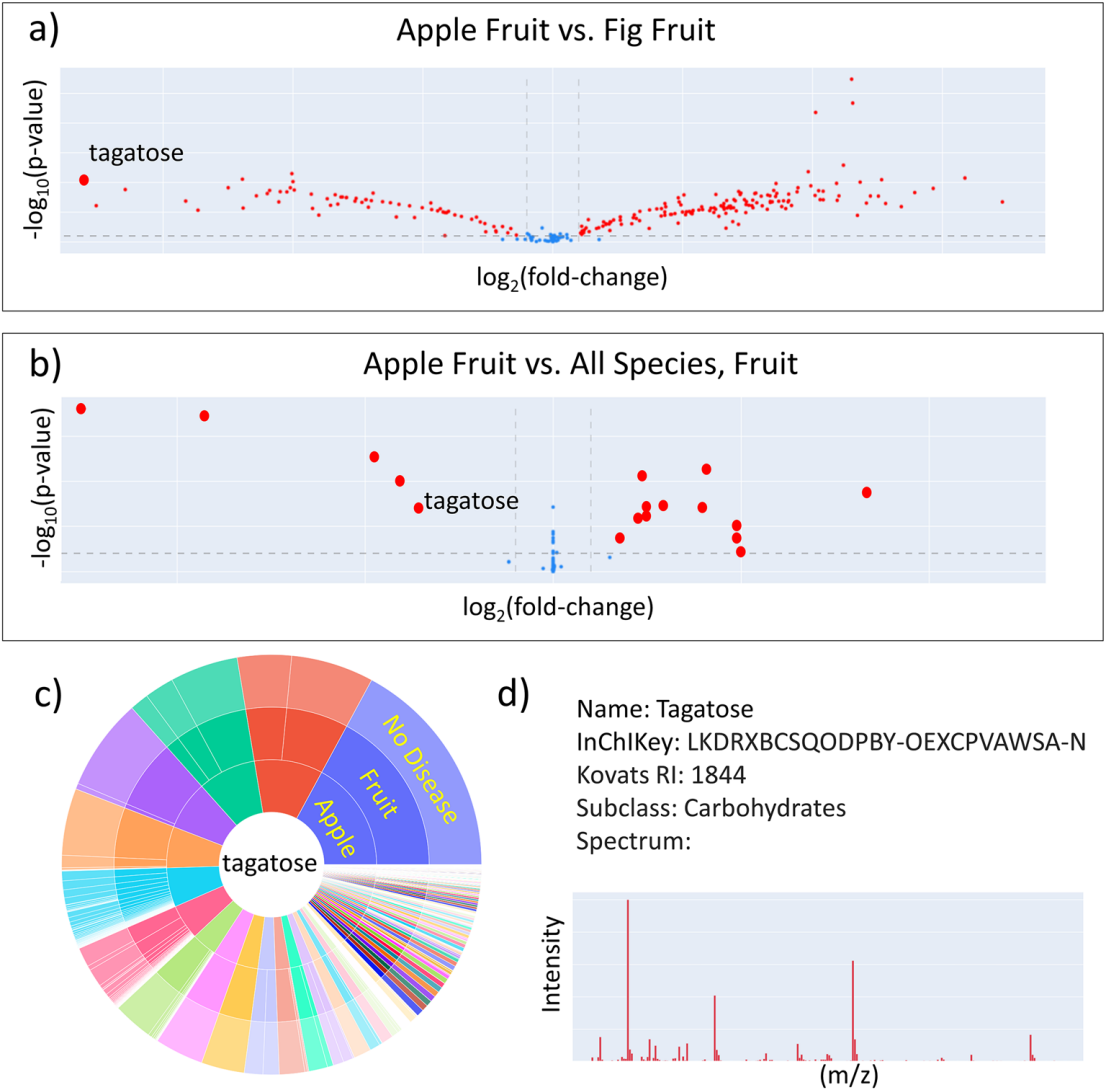
Queries in BinDiscover give novel biological insights. **a** Comparing the metabolome of a specific organ across two different species, here: apple vs. fig fruits, yields many differences. **b** Comparing that specific organ (apple fruit) against the same organ of all species constrains overall differences to a few metabolites. **c** One differential apple metabolite, tagatose, was then queried and found to be the most abundant in apple fruits compared to all other species/organ combinations across the metabolome database. **d** Chemical information for tagatose is then given as mass spectrum, quantification mass, international chemical identifier, retention index and chemical class ontology

Metabolomics is a hypothesis generating tool. Databases must prove their usefulness by serving specific queries. We here provide four use cases to highlight how biologists or biomedical scientists might use the BinDiscover webtool. To enable rapid exploration of the metabolome data on differences between species, organs and diseases, users define ontologically grouped differential analysis on biological metadata, or explore data from a compound-centric pool. The webtool relies on commonly accepted statistics and clear graphics to obtain rapid insights into major metabolic differences in biological comparisons (Fig. 4).

We first envisioned a *nutritional researcher* exploring this tool. Food metabolomes and dietary biomarkers are increasingly recognized as important contributor to disease [21, 22]. As a starting point, a researcher might wonder why “an apple a day keeps the doctor away”? The user might choose to compare an apple to any other fruit, in this case a fig (Fig. 4a). Such a comparison is valid and produces a large amount of information comparing these two fruits. When hovering over the online graph (Fig. 4a), each dot represents an individual compound. Tagatose is highlighted here as the metabolite that showed the largest difference in apple over fig fruits. At this point, the user might want to increase the query and compare apple fruits to all fruits in the BinDiscover database (currently 26 fruits). In this way, researchers find out which metabolites are uniquely increased, or decreased, in apples compared to all other fruits. Interestingly, this query still showed tagatose to be found in higher levels in apples than in other fruits (Fig. 4b), with notably fewer total metabolic differences compared to the differential analyses of the apple/fig pair. The online data tables that correspond to the visual charts show all differential metabolites and guide users to compound-specific follow up queries. Here, the envisioned nutritionist user would find a sunburst diagram and chemical metadata (Fig. 4c, d). The sunburst diagram shows that indeed, tagatose showed the highest intensity in apple fruits across all species/organ/disease metadata combinations. Such finding may be interesting because tagatose, despite containing 92% of the sweetness of sucrose, provides only approximately 1/3 of the calories compared to sucrose [23]. Moreover, tagatose does not increase insulin in patients with Type-2 diabetes [24]. Researchers might use this finding as starting point for additional research, e.g. apple genomic tools to increase tagatose contents in other fruits or even in apple cultivars.

### Case study 2—cancer metabolism

**Fig. 5:**
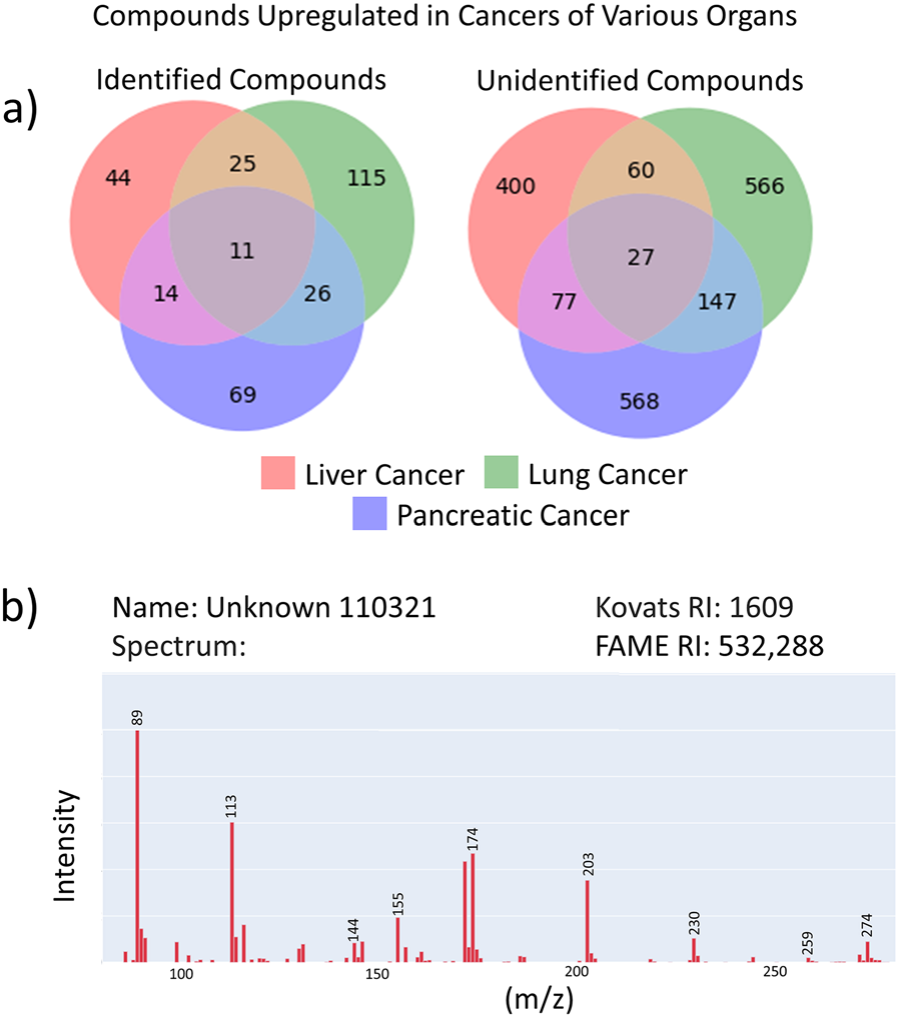
Sequential queries extract unknown metabolites associated with cancer metabolism. **a** Integrating results from three BinDiscover queries comparing liver, lung and pancreas cancer studies with and without cancer yields three sets of compounds. Results are separated here between identified and unknown compounds. **b** BinDiscover gives spectra and chemical metadata to enable chemists to utilize unknown compounds in their own studies, either for targeting these compounds in their own studies or for compound identification. Here, unknown 110,321 is displayed.

Next, we envisioned a *cancer biologist* interested using BinDiscover. Here, we highlight how repeatedly utilizing the BinDiscover differential analysis tool empowers isolating both identified and unknown compounds that distinguish cancer metabolic phenotypes from corresponding non-malignant analogs, and how different cancer cells and tissues would reveal specific alterations that are not prevalent in other cancers. Specifically, for proof of principle, we obtained three metadata combinations for lung, liver and pancreas cancers, each compared against their non-malignant counterparts. In each utilization, we obtained a set of compounds. By taking the intersection of the resultant sets, a cancer biologist may find compounds that are differentially regulated in all cancer types (Fig. 5a), and compounds that would be specific for each cancer type. We found 11 identified compounds that intersected with all cancers, such as increases in glutamine, dehydrated glutamine, *n*-acetylglutamate, and methylmalonic acid (Additional file 1: Data S1). These compounds can be associated with tricarboxylic acid (TCA) cycle activity, specifically for anaplerotic reactions supplanting carbon into the TCA cycle. For example, excess glutamine is known to be heavily used in cancerous cells in particular via glutamine dehydrogenase to generate glutamic acid, which is then converted to alpha-ketoglutarate [25]. Similarly, the branched-chain amino acid degradation product methylmalonic acid is converted to the TCA metabolite succinyl-CoA in an anaplerotic reaction, as cancer cells are deprived of mitochondrial acetyl-CoA due to lowered activity of pyruvate dehydrogenase. Another typical cancer biomarker found by this combined BinDiscover differential analysis was increased pyrophosphate, which is associated with increased kinase activity and cell growth [26]. Additionally, we explored apparent compounds that might distinguish the three cancer types investigated here (Additional file 1: Data S1).

For example, in pancreatic cancers we observed increased amounts of all four forms of tocopherol, also known as Vitamin E. Vitamin E has been proposed to be associated with decreased pancreatic cancer risk, in opposite to our findings [27]. We also noticed several dipeptides to be increased specifically in pancreatic cancer studies, such as cystine, homocystine, and dialanine (Additional file 1: Data S1), indicating enhanced import of peptides as supplement nutrients or increased proteolysis. For lung cancer studies, we noted specific increased levels in alpha-keto acids such as 2-ketoisocaproic acid and 2-ketoisovaleric acid along with corresponding alpha-hydroxy acids like 2-hydroxyvaleric acid and 2-hydroxyglutaric acid (Additional file 1: Data S1). These compounds are usually associated with increased use of amino acid degradation. Lung cancer studies were also marked by elevated acetylations, including *N*-acetyl-glycine, -mannosamine, -serine, -aspartate and -putrescine (Additional file 1: Data S1). The latter two compounds have previously been proposed as biomarkers of lung cancer progression [28, 29]. For liver cancer, the most apparent specific trend that was absent in prostate- or lung cancer studies was the abundance of glycolytic intermediates galactose-6-phosphate, fructose-6-phosphate, fructose-1,6-bisphosphate, 3-phosphoglycerate, 2-phosphoglycerate, and phosphoenolpyruvate, along with the pentose phosphate cycle metabolite ribulose-5-phosphate, and generic sugar phosphates inositol-4-monophosphate and *N*-acetylglucosamine-6-phosphate (Additional file 1: Data S1). An increased glycolytic flux is not only well-known for liver cells [30] but also a generic hallmark of cancer and, according to studies available in BinBase, much elevated in liver cancers compared to lung- or pancreatic cancers. Apart from classic known metabolites, chemists and metabolomic researchers might assist cancer researchers in finding novel clues towards metabolic dysregulation in cancer. Here, we found more than 1,500 unidentified compounds that were specific for the three cancer types, and 27 unknown compounds that were commonly differentially regulated in all cases (Additional file 2: Data S2). The chemical metadata for a randomly chosen example from the 27 common dysregulated compounds, unknown 110,321, is shown in Fig. 5b. As BinBase gives both spectra, quantification ions and retention indices, other metabolomics researchers can readily use that information to target these unidentified cancer biomarkers in their studies. Secondly, spectra of novel biomarkers serve as starting point for compound identification. Compound 110,321 shows a range of even-numbered fragment ions such as m/z 144, 172, 174, which are typical of primary amines, plus high m/z ion clusters around m/z 274 and m/z 230 which also point to the presence of nitrogen moieties. The spectrum lacks m/z 117, a typical fragment for carboxylic acids and sugars. The retention indices reveal a compound that has a boiling point similar to other amino acids, and hence, compound 110,321 can be classified as a primary amine with additional functional groups such as a secondary amine. With chemical ionization/accurate mass spectrometry, the full structure would then become identifiable [31].

### Case study 3—diversity of bacterial metabolism

**Fig. 6:**
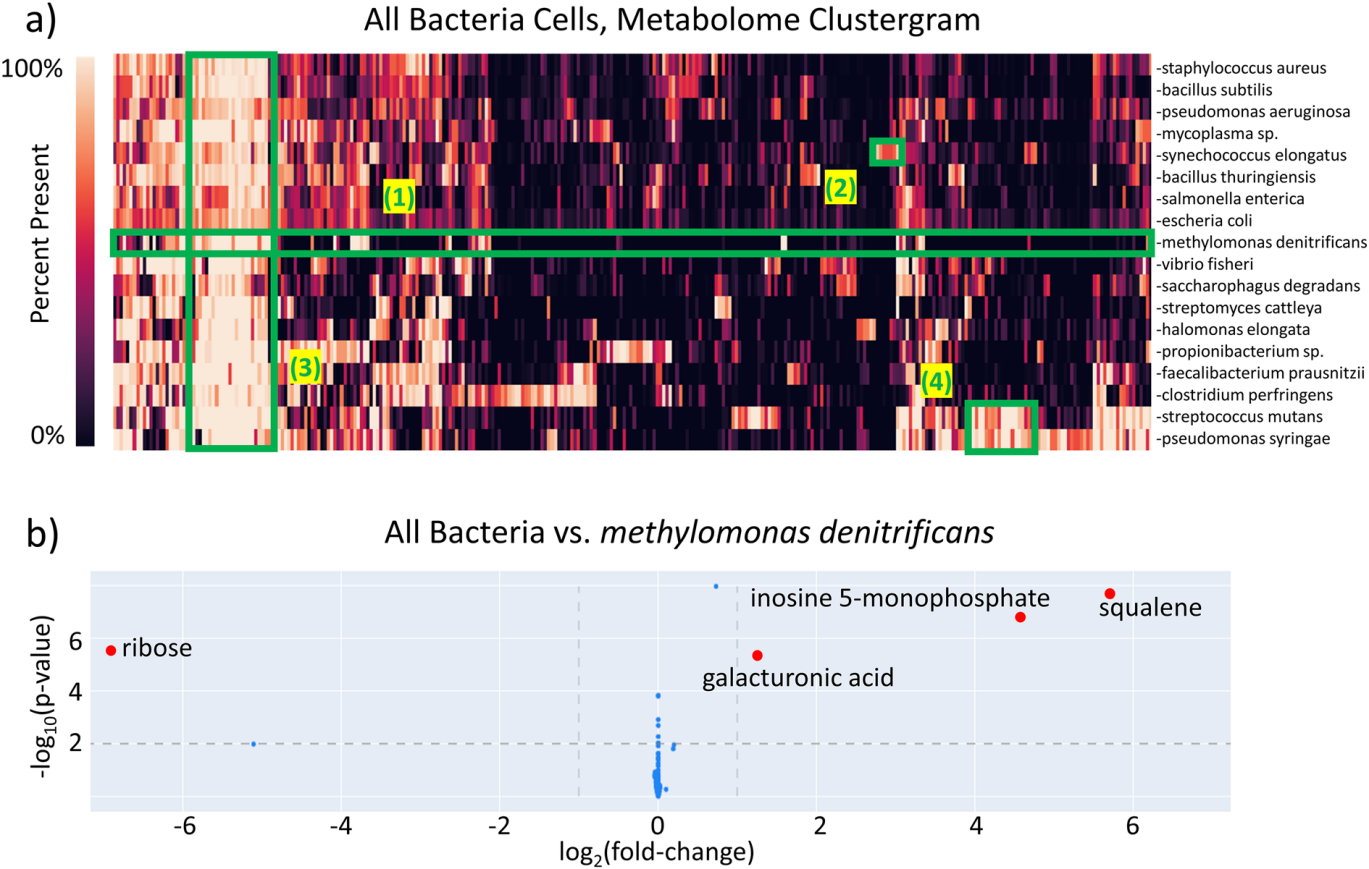
Comparison of the gas chromatography metabolomes of bacteria in BinDiscover. **a** A heatmap of all metabolites in BinDiscover against all available bacteria species. Matrix entry color is determined by percent presence of that metabolite in that species. Four regions of interest (1)–(4) are highlighted in green and discussed in the text. **b** A differential comparison of metabolomic abundances in bacteria species against the methane-metabolizing species *Methylomonas denitrificans*

A *microbiologist* might use BinDiscover to study bacterial metabolism across species, for example, as background for synthetic biology supplanting traditional synthetic routes [32]. Likewise, the gut microbiome is gaining focus as the source of many endogenous metabolites as well as the origin of phenotypes in pharmaceutical testing [33]. The diversity of potential of bacterial metabolic function is of interest, and we therefore generated a clustered heatmap as phylo-metabolomic tool in BinDiscover (Fig. 6). These phylo-metabolomic heatmaps utilize the chemotaxonomic presence of all detected metabolites (in columns) against the specified combination of taxa (in rows) using hierarchical clustering.

Such heatmaps can be used to delineate specific outlier species, as shown for highlighted section #1 in Fig. 6a for *Methylomonas denitrificans* which uses methane metabolism as its carbon source. A detailed BinDiscover comparison of this species against all other bacteria (Fig. 6b) revealed much elevated production of squalene [34] and inosine-5-phosphate concomitant with reduced ribose biosynthesis. Section #2 in Fig. 6a highlighted a cluster of compounds that were unique to *Synechococcus elongatus*, a blue-green photosynthetic algae, that produces the pigment trans-phytol in addition to various alkanes that were absent in all other bacteria in BinDiscover. Section #3 contained ubiquitously present metabolites such as fatty acids, amino acids, and nucleic acids, which therefore did not contribute to bacterial classifications. Finally, Section #4 marked a section of metabolites that linked the human mouth bacterium *Streptococcus mutans* and the plant pathogen *Pseudomonas syringae*. Observed metabolites included tryptamine and indole-3-acetate, which have been included in publications studying the host-pathogen relationship [35, 36]. In general, the diversity present in these bacterial metabolomes reflects the niches that are to be expected [37]. We focus on biological concepts in the case studies presented here because we BinDiscover itself is intrinsically informatics-oriented. An additional case study that is more oriented toward cheminformatics where we showcase the relevance of unknown compounds is shown in Additional file 3: Fig. S4.

## Discussion

BinDiscover effectively enables rapid meta-analysis of metabolomics information with the objective of ease of use for biological scientists, focusing on both capability and breadth of metabolome coverage. However, post-hoc retrieval and harmonization of biological sample metadata were challenging. To our knowledge, there are scant examples of usable interfaces that correctly map biological study designs, covering not only species and organs, but also treatments, time courses or disease phenotype dimensions of study designs. Hence, two of the most important issues concerning to sample metadata were the inconsistency of metadata terminology used when capturing biology study information in our miniX study design DB and the omission of fine-grained biological study design details. Inconsistent metadata terminology describes the informality by which samples were labeled by biologists who were sending studies to the UC Davis West Coast Metabolomics Center over the past 18 years. While for domain experts, a word such as “C57BL/6” might sufficiently describe a specific mouse wildtype, even for this classic example there are different laboratory strains such as B6J (or B6/J) for mice from the Jackson laboratory, and similar strain variants from other laboratories. The same is true across other biological domains, from cell types to fine grained descriptions of tissues and organs. Closely related to this omission of details was the difficulty to capture the essence of biological studies, such as the use of specific gene knockouts or drug treatments. Information was sometimes delivered by biologists in text formats and through sample lists, but usually domain-specific acronyms were used that were intractable to compile retrospectively throughout the diversity of 2000 different studies in our GC/MS database. It is worth mentioning that the case studies presented emphasize BinDiscover’s biological applications as a complement to its intrinsic nature as an informatics tool. Of course, BinDiscover has applications beyond those presented here, and we offer a cheminformatics-based discovery of unknown-unknowns in the GC/MS metabolome via Additional file 3: Fig. S4.

An alternative approach to programmatically capture study design details might use named-entity recognition combined with NoSQL/GraphDB records. An entity recognition system might start with a vocabulary of known ontologies, but would need to be capable to expand an internally consistent vocabulary to capture arbitrary descriptions. While a graph approach allows for robust and dynamic descriptions of samples and their relationships, the named-entity recognition avoids problematic curation. Yet, a graph-based interface would present significant complexities for users, especially biologists who are asked to submit their study information. Initial efforts led to frustrations and overwhelmed potential users. An alternative approach to capture study metadata is to pre-define motifs of study designs and coerce study design details into those motifs. While many fine-grained study details (and, hence, sample metadata resolution) get lost in coarse motif-based GUI forms, such tools may dramatically simplify the procedure for biological clients. While not comprehensive, reducing the burden on researchers can dramatically increase the likelihood that individuals will contribute these details when using metabolomics (or other -omics) services.

Importantly, ontologically grouped differential analysis offers important quantitative results that simple presence/absence analysis ignores. For example, sucrose is present and detected at low amounts by untargeted GC–MS metabolomics in human blood. However, it would be wrong to conclude that sucrose is a major constituent in human samples, compared to plant samples. Here, semi-quantitative assessments are possible in GC–MS based metabolomics for two reasons: (a) Electron ionization at 70 eV is standardized in GC–MS for 60 years, and it does not suffer from suppression by co-eluting compounds, unlike electrospray processes used by LC–MS/MS. An exception to this rule is the vicinity of compounds that exceed peak saturation, e.g. urea in urine. (b) Extraction, derivatization, injection, detection and data processing methods at UC Davis have been standardized to assure that chromatograms were never overloaded (i.e. avoiding peak saturations), but also never blank (ensuring that the most abundant peaks in specific samples were reaching detector saturation). Hence, semi-quantification was assured by both data acquisition and data processing procedures, including using the exact same concentration of (fatty acid methyl ester) internal standards over the past 18 years. Nevertheless, of course despite these precautions, quantitative results must be interpreted with care. For example, comparisons across organs may include biofluids versus tissues, i.e. different units of biomass. In addition, different solvent extraction efficiencies across different tissues or biofluids may introduce bias. Finally, abundances can be underestimated when detector saturation occurs during the coelution of very high abundance and very low abundance compounds. Hence, quantitative comparisons that yield large fold-change differences can be interpreted with higher confidence than small differences. Indeed, one of the goals of ontologically grouped differential analysis is to conservatively minimize the fold changes for each compound among the set of requested organs in order to increase confidence in these quantitative findings. However, for biological metadata combinations with few samples and few studies, quantitative comparisons are less robust than for differential analyses for which there were thousands of sample data available in BinBase.

Additionally, BinDiscover is built on top of a snapshot of BinBase data as they were in December 2021. As BinBase continues to expand, novel compounds get added. For example, in November 2022, we reliably detected the presence of carboxymethylcysteine (Additional file 3: Fig. S3) for the first time, in a study analyzing bovine muscle tissues, treated with inhibitors against oxidative phosphorylation complexes. Compounds in BinBase (such as carboxymethylcysteine) might have been present infrequently or at low abundance before their successful induction into BinBase. To overcome such metadata incongruencies, BinDiscover focuses on high-level analyses of species and organ queries using ontological differential analysis, sunburst diagrams, and phylo-metabolomic trees. Users obtain the number of samples for each query and metadata combination with the notion that the estimation of median metabolite levels gets more robust the more samples are included in comparisons. Even more specific metadata comparisons may provide insights into metabolic differences if users focus on compounds with sufficiently large fold changes.

BinDiscover aims at hypothesis-generating and data exploration. We are motivated to discover unexpected findings, and, contextually, are relatively unconcerned about false-positives (type I error). We do note that it would be common to reduce the error rate using the Benjamini–Hochberg procedure or similar approaches, but rationally avoided this step because we were interested in increasing the recall of explorable findings. Likewise, we wanted to explore approaches for finding generalized groups of samples in parallel with exploring the data themselves, so we here introduce the ontologically grouped differential analysis. Similarly, we do not use Fisher’s method to aggregate p-values when combining metadata combinations, because we compare completely different hypotheses in each pairwise comparison, using ontologically grouped differential analysis.

Future versions of BinDiscover may become incrementally updated by data from new studies including from public contribution. A related tool, ADAP-KDB, perpetually retrieves and updates a user-explorable consensus library of spectra from the MetabolomicsWorkbench [38]. ADAP-KDB does not use static snapshots and focuses on a community-contributed source of data, but it is clearly spectrum-centric and assisted by the de-facto standards in GC–MS. We hope that there will be community-wide efforts to further standardize standard operating procedures for metadata definitions, sample extraction, data acquisition, and data processing to confidently include broader contributions from the community into GC-Binbase.

It is critical that meta-analysis systems for metabolomics focus on *samples, not on studies*. In this way, metadata of samples can be repurposed for new biological comparisons, conducted from a library of analyzed samples. At current, meta-analysis often relies on combining studies that had approximately the same intention, which dramatically reduces the ways in which data can be re-used. As a part of this grand unification of metabolomics data, we hope that standardization in metabolomics will improve. The inclusion of *internal standard kits* as matrix spikes into samples before extraction could serve as a check of instrument state as well as allow for semi-quantitative, on-the-fly calibrations that would dramatically improve the level of confidence in sample-to-sample integration.

## Conclusions

BinDiscover is a webtool based on a 156,000 sample GC–TOF database that has accumulated data since 2005. We curated this dataset by removing samples that failed quality control checks, imputing missing values, and mapping the metadata as well as identified metabolites to established ontologies. We showed that our webtool enables rapid hypothesis generation and trend extraction in order to transform machine-sized databases into human-sized, actionable simplifications. Our tool provides components that enable the examination of large swaths of data simultaneously as well as the ability to focus on individual compounds. We enable the comparison of multiple types of species and organs using chemotaxonomy trees and ontologically grouped differential analysis, but also the visualization of single compounds with sunburst diagrams or chemical metadata. One novel approach to data analysis, ontologically grouped differential analysis, uses external ontologies, such as the NCBI species taxonomy or MeSH hierarchy, to create groups of samples that match generic terms. The logic of ontologically grouped differential analysis can be applied to arbitrary metadata or features, as long as a corresponding ontology exists, so we believe that it has applicability for other -omics as well. Hence, queries can be grouped along the ontology axes, for example, to compare “rodent blood” against “human blood” or similar broad groupings. Metabolomics is now mature enough to empower re-using data deposited in large scale databases derived from standardized methods, with the explicit aim to perform meta-analyses across disparate studies. We strongly emphasize the importance of metabolome standardization initiatives that are critically needed for cross-study and cross-species data comparisons. Indeed, this type of sample-centric data collection could form training sets for large scale phenotype-predicting machine learning models. We found that one of the most challenging aspects in the creation of this metanalysis tool was curating and harmonizing the swaths of metadata submitted by biologist clients. We envision working toward simplified, yet powerful metadata capture systems.

## Supplementary Information

**Additional file 1.** Identified compounds upregulared in cancer.

**Additional file 2.** Unknown compounds upregulated in cancer.

**Additional file 3.** Supplemental Figures and Tables.

## Data Availability

All code is available at https://github.com/metabolomics-us/bindiscover. The complete distributions are available at https://zenodo.org/record/7982901. The derived data are available via https://bindiscover.metabolomics.us. API documentation is available at https://metabolomics-us.github.io/bindiscover/.

## Abbreviations

GC: Gas chromatography
TOF: Time of flight
MS: Mass spectrometry
GNPS: Global Natural Products Social Molecular Networking
ReDU: Reanalysis of data user
HMDB: Human Metabolites Database
COCONUT: COlleCtion of Open Natural ProdUcTs
API: Application programming interface
OGDA: Ontologically grouped differential analysis
AWS: Amazon Web Services
GUI: Graphical user interface
FAME: Fatty acid methyl ester
NCBI: National Center for BioInformatics
MeSH: Medical Subject Headings
MAR: Missing at random
MNAR: Missing not at random
MDC: Metadata combination
TCA: Tricarboxylic acid
