# Supplementary material for: The BinDiscover database: a biology-focused meta-analysis tool for 156,000 GC–TOF MS metabolome samples
Source: J Cheminform. 2023 Jul 20;15:66. doi: 10.1186/s13321-023-00734-8 (PMC10359220; doi:10.1186/s13321-023-00734-8)
Supplement: Supplementary file 3 — Additional file 3. Supplemental Figures and Tables. [file 13321_2023_734_MOESM3_ESM.docx]

**Additional Figures and Tables for**

**The BinDiscover database: a biology-focused meta-analysis tool for 156,000 GC-TOF MS metabolome samples** Parker Ladd Bremer^1^, Gert Wohlgemuth^2^, and Oliver Fiehn^2*^

^1^ Department of Chemistry, University of California, Davis, California 95616, U.S.A.

^2^ West Coast Metabolomics Center for Compound Identification, UC Davis Genome Center, University of California, Davis, California 95616, U.S.A

Keywords: metabolomics, gas chromatography, mass spectrometry, meta-analysis, ontologies,

(*) Corresponding author: [ofiehn@ucdavis.edu](mailto:ofiehn@ucdavis.edu)

**
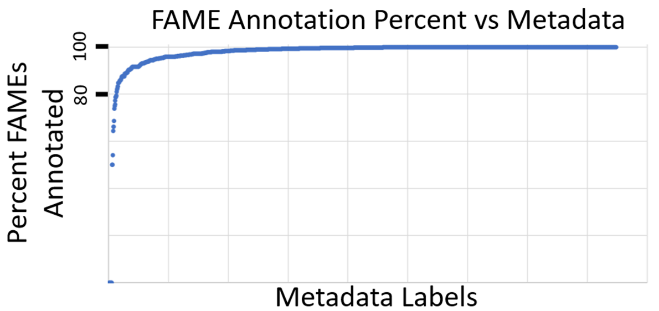
**

**Figure S1:**

**Frequency of FAME detections across all BinBase study samples (1,696 metadata triplet combinations).** Samples were removed if they belonged to metadata combinations with a FAME annotation frequency less than 80%.

**
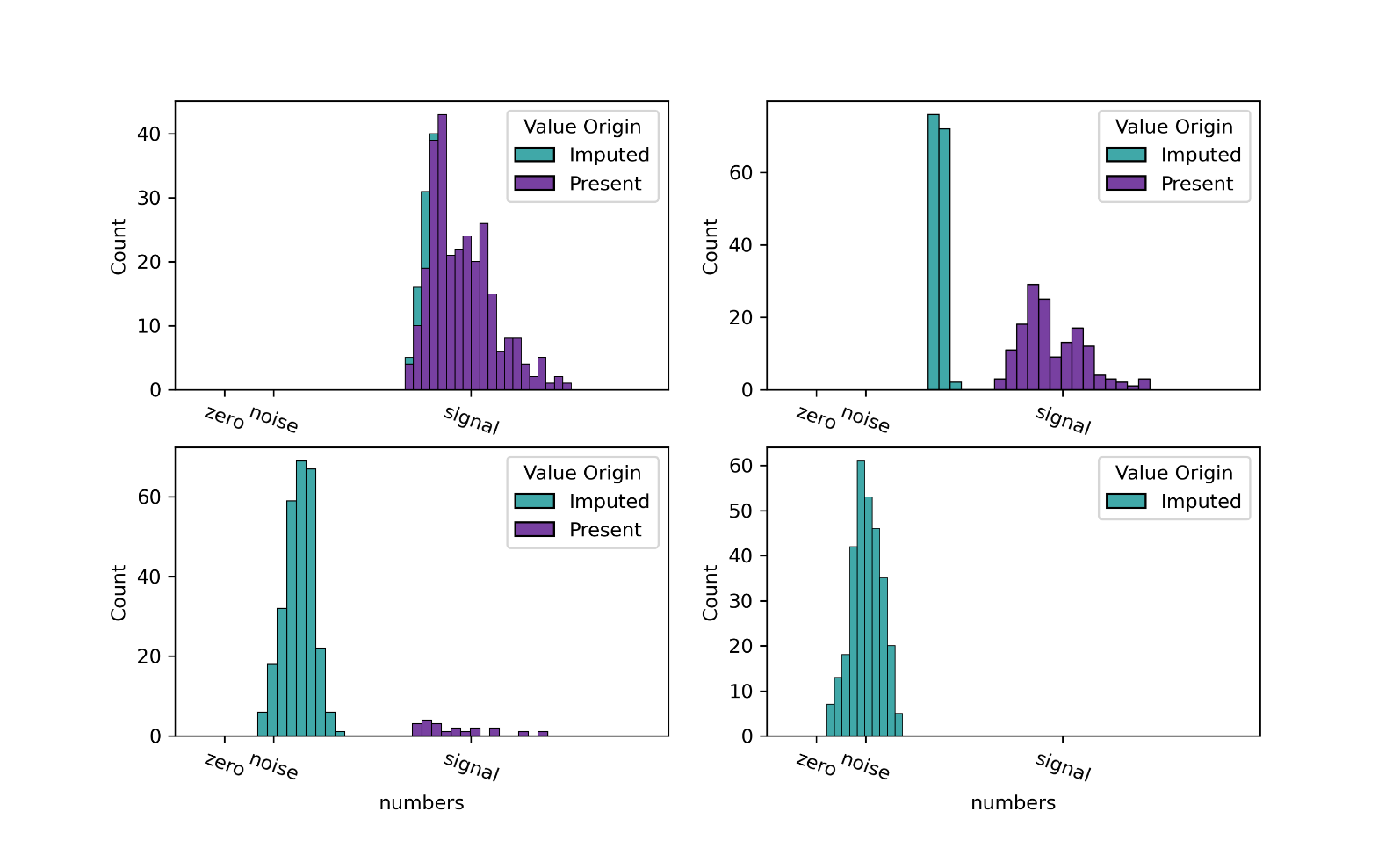
**

**Figure S2: Concept graphs of imputed data (to replace missing data) versus experimentally detected data.** These concept graphs use simulated data, not data obtained from BinBase, to illustrate four different scenarios of how missing metabolite data in BinBase metadata combinations might be overcome.

(a) **Top left.** Data missing at random might have been missed by experimental causes, such as data processing thresholds or instrument malfunctions.

(b) **Bottom left.** Data missing not at random, but with many missing data and few detected data for metadata combinations. Examples could be for metabolites that were generally found at low levels and for which experimental limits-of-detections caused data missingness in many samples, but not in all samples. Another cause for data missingness in this scenario is metabolites that are synthesized or detected only in specific conditions, such as pharmaceutical drugs in human plasma that may be found in high levels in some subjects, but very low or absent in most others.

(c) **Top right**. Data missing due to unexplained differences in study design parameters that impact absence or presence of metabolite not at random. Example for such rare cases could be different animal feeds used in rat plasma metabolome studies, or age-related metabolites that were present in one study but not in another. Typically, such not-at-random gross missingness might only be found in metadata combinations that have a small total sample count.

(d) **Bottom right.** Metabolites that were completely absent in specific metadata combinations. Yet, to compute fold-changes for differential analyses, instrument noise levels are used to impute missing data around defined variance.

**Figure S3: S-carboxymethylcysteine, a compound detected in November 2022 in bovine skeletal muscle and automatically added to BinBase.**

1. Experimentally observed spectrum (red) compared to reference library spectrum (blue) and identified by both mass spectral and retention index similarity.
2. Chemical compound information for S-carboxymethylcysteine, including the international chemical identifier hash key (InChI), the BinBase identifier, the FAME-based retention index 626424, the Kovats calculated retention index, and the quantification mass.


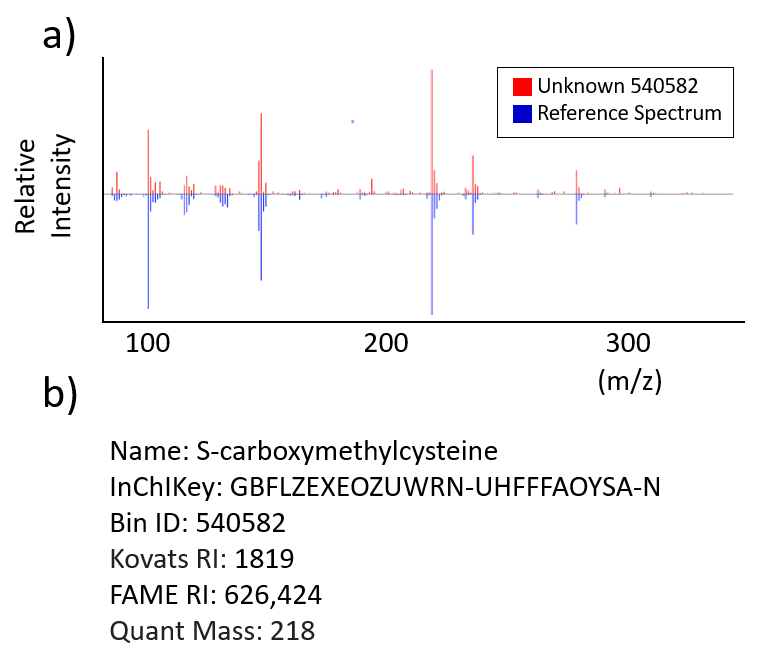


**
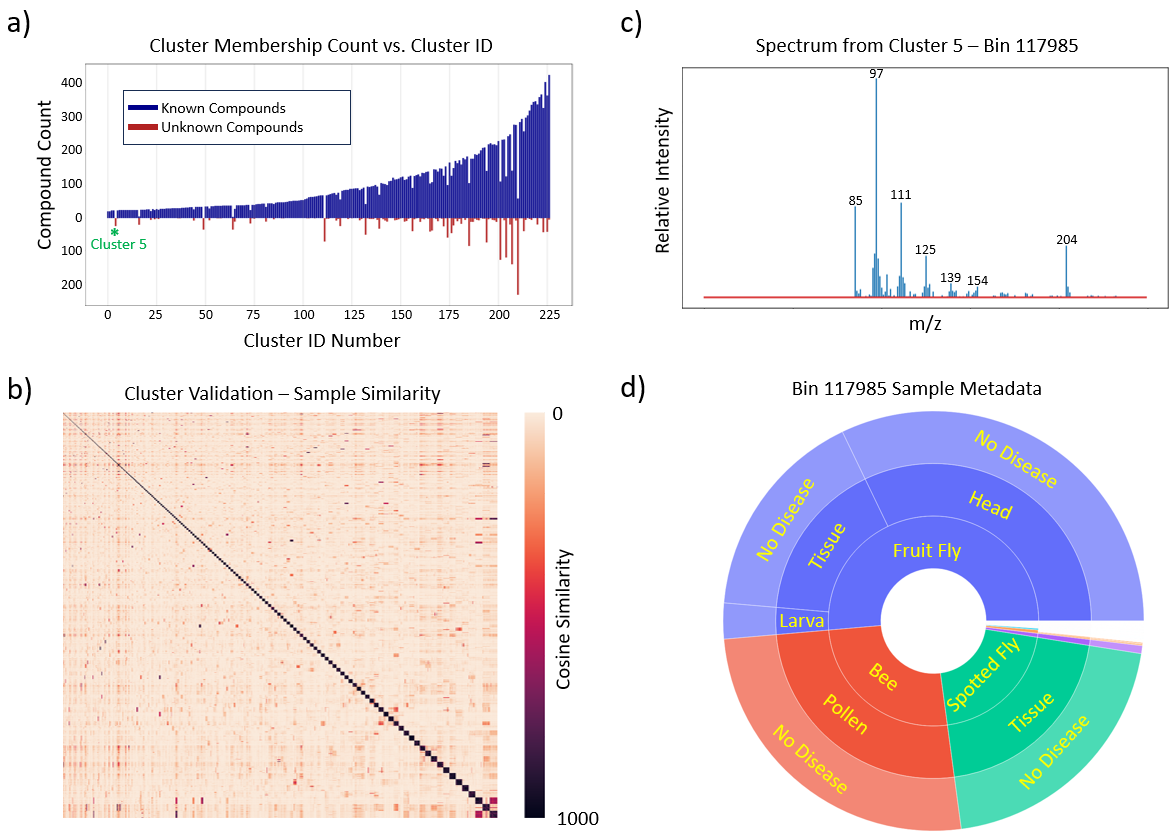
**

**Figure S4: Discovering Unknown Unknowns in the GC/MS metabolome..**

1. The spectra of unknowns in BinDiscover and NIST17 GC/MS were clustered using DBSCAN with a minimum cluster membership of 25 and a cosine similarity of 950 (set high to identify distinct archetypes). This yielded ~225 clusters containing both known and unknown compounds. It was observed that certain clusters were comprised (almost) entirely of unknown spectra, which means that there were no similar spectra in NIST17. If we assume that NIST17 is a comprehensive authority on GC/MS spectra, then the compounds associated with these spectra remain outside of those measured and could not be identified using a library-retrieval approach.
2. Clusters were validated by visualizing the similarity between spectra when spectra were sorted by cluster membership. Visually distinct squares arose along the diagonal, which indicates high cluster purity.
3. An example bin’s spectrum from cluster 5 is visualized.
4. The associated metadata for the same bin is visualized. This compound appears to originate in insects that interact with fruiting plants.

**Table S1:**

Example metadata combinations associated with ontological queries, used in Figure 1.

| From or to | Metadata Triplet | Sample Count |
| --- | --- | --- |
| from | homo sapiens - Pancreas - No Disease | 217 |
| from | homo sapiens - Duodenum - No Disease | 183 |
| from | homo sapiens - Liver - No Disease | 471 |
| to | lactobacillales - Cells - No Disease | 16 |
| to | pseudomonas syringae - Cells - No Disease | 16 |
| to | synechococcus elongatus - Cells - No Disease | 414 |
| to | vibrio fischeri - Cells - No Disease | 13 |
| to | saccharophagus degradans - Cells - No Disease | 55 |
| to | helicobacter pylori - Cells - No Disease | 24 |
| to | salmonella enterica - Cells - No Disease | 66 |
| to | chromobacterium - Cells - No Disease | 24 |
| to | ralstonia eutropha - Cells - No Disease | 17 |
| to | streptomyces cattleya - Cells - No Disease | 11 |
| to | bacillus subtilis - Cells - No Disease | 88 |
| to | staphylococcus aureus - Cells - No Disease | 78 |
| to | mycoplasma - Cells - No Disease | 12 |
| to | faecalibacterium prausnitzii - Cells - No Disease | 66 |
| to | synechococcus - Cells - No Disease | 27 |
| to | clostridium perfringens - Cells - No Disease | 36 |
| to | streptococcus mutans - Cells - No Disease | 18 |
| to | pseudomonas aeruginosa - Cells - No Disease | 163 |
| to | escherichia coli - Cells - No Disease | 1313 |
| to | propionibacterium - Cells - No Disease | 24 |
| to | clostridium - Cells - No Disease | 36 |
| to | methylomonas denitrificans - Cells - No Disease | 12 |
| to | halomonas elongata - Cells - No Disease | 11 |
| to | bacillus thuringiensis - Cells - No Disease | 170 |

**Table S2:**

Significant compounds resulting from the query in Table S1

| Compound Name | InChIKey | log_2_(fold-change) | p-value |
| --- | --- | --- | --- |
| zymosterol | CGSJXLIKVBJVRY-XTGBIJOFSA-N | -4.1 | 9.01E-20 |
| ascorbic acid | CIWBSHSKHKDKBQ-JLAZNSOCSA-N | -7.4 | 1.38E-40 |
| 5-hydroxy-3-indoleacetic acid | DUUGKQCEGZLZNO-UHFFFAOYSA-N | -6.4 | 7.19E-55 |
| (5E)-isovitamin D3 | LMBGVVOJTGHJNP-FVUVGDFOSA-N | -3.2 | 8.02E-03 |
| docosahexaenoic acid | MBMBGCFOFBJSGT-KUBAVDMBSA-N | -8.8 | 1.35E-94 |
| cholesterone | NYOXRYYXRWJDKP-GYKMGIIDSA-N | -4.9 | 6.39E-16 |
| hexadecylglycerol | OOWQBDFWEXAXPB-UHFFFAOYSA-N | -3.0 | 9.32E-30 |
| tocopherol gamma- | QUEDXNHFTDJVIY-DQCZWYHMSA-N | -2.3 | 1.21E-37 |
| epicholestanol | QYIXCDOBOSTCEI-FBVYSKEZSA-N | -7.1 | 4.38E-50 |
| campesterol | SGNBVLSWZMBQTH-PODYLUTMSA-N | -4.7 | 1.32E-19 |
| 2-monoolein | UPWGQKDVAURUGE-KTKRTIGZSA-N | -2.7 | 9.75E-27 |
| hypotaurine | VVIUBCNYACGLLV-UHFFFAOYSA-N | -4.2 | 1.54E-33 |
| D-erythro-sphingosine | WWUZIQQURGPMPG-KRWOKUGFSA-N | -1.2 | 1.87E-03 |
| N-methylglutamic acid | XLBVNMSMFQMKEY-BYPYZUCNSA-N | -4.1 | 3.60E-21 |
| arachidonic acid | YZXBAPSDXZZRGB-DOFZRALJSA-N | -5.4 | 5.61E-24 |
